# Association between body mass index and severe infection in older adults with microscopic polyangiitis: a retrospective cohort in Japan

**DOI:** 10.1186/s12877-021-02123-y

**Published:** 2021-03-09

**Authors:** Hirokazu Sugiyama, Makoto Yamaguchi, Takayuki Katsuno, Shiho Iwagaitsu, Hironobu Nobata, Hiroshi Kinashi, Shogo Banno, Masahiko Ando, Yoko Kubo, Takuji Ishimoto, Yasuhiko Ito

**Affiliations:** 1 Department of Nephrology and Rheumatology, Aichi Medical University, 1-1 Karimata, Yazako, Nagakute, Aichi 480-1195 Japan; 2 Data Coordinating Center, Department of Advanced Medicine, Nagoya University Hospital, Nagoya, Japan; 3 Department of Preventive Medicine, Nagoya University Graduate School of Medicine, Nagoya, Japan; 4 Department of Nephrology and Renal Replacement Therapy, Nagoya University Graduate School of Medicine, Nagoya, Japan

**Keywords:** Antibodies, Antineutrophil cytoplasmic antibody-associated Vasculitis, Body mass index, Infection

## Abstract

**Background:**

Although previous studies have evaluated risk factors for the incidence of severe infection in patients with antineutrophil cytoplasmic antibody-associated vasculitis (AAV), the relationship between body mass index (BMI) and severe infection in AAV has not been elucidated. We hypothesized that older adults with AAV and a low BMI would be at a higher risk of infection. We therefore investigated the association between underweight status at AAV diagnosis and subsequent occurrence of severe infection in older adults with AAV.

**Methods:**

This single-center retrospective cohort study included 93 consecutive older adults with microscopic polyangiitis (MPA) treated at the Aichi Medical University Hospital in Japan between 2004 and 2018. The relationships between BMI at diagnosis and subsequent first severe infection were assessed using multivariate Cox proportional hazards models. The cumulative probability of the development of the first severe infection was calculated using the Kaplan-Meier method and the log-rank test. The level of statistical significance was set at *P* <  0.05.

**Results:**

During the median follow-up period of 19 (6–53) months, 29 (31.2%) patients developed at least one severe infection. Older age (adjusted hazard ratio [HR] = 2.02, 95% confidence interval [CI]: 1.14–3.52, per 10 years; *P* =  0.016), low BMI (< 18.5 kg/m^2^ compared with normal BMI [18.5–23.0 kg/m^2^], adjusted HR =  2.63, 95% CI: 1.11–6.19; *P* =  0.027), and use of methylprednisolone pulse therapy (adjusted HR = 2.48, 95% CI: 1.07–5.76; *P* =  0.034) were found to be significant predictors of severe infection.

**Conclusions:**

Low BMI was associated with a higher risk of severe infection in older adults with MPA, suggesting that careful management may be required to prevent this complication in this vulnerable group. Further studies are needed to elucidate the optimal treatment strategy for these patients.

**Supplementary Information:**

The online version contains supplementary material available at 10.1186/s12877-021-02123-y.

## Background

Antineutrophil cytoplasmic antibody (ANCA)-associated vasculitis (AAV), which includes microscopic polyangiitis (MPA), granulomatosis with polyangiitis (GPA), and eosinophilic granulomatosis with polyangiitis (EGPA), is characterized by necrotizing vasculitis of the small vessels and high ANCA-positivity [1, 2].

Recently, advances in the treatment strategy for AAV have helped reduce fatal organ damage [3–7]. However, as compared to the general population, AAV patients show markedly high rates of severe infection, which is an important complication associated with morbidity and mortality in this group [8]. Previous studies have identified several risk factors for the development of infection in AAV, including chronic use of high-dose glucocorticoids (higher cumulative exposure), use of immunosuppressive agents, older patient age, leukopenia, lymphopenia, and kidney dysfunction [8–18].

Body mass index (BMI) has been regarded as an indicator of nutritional issues, and malnutrition and overnutrition can lead to underweight and overweight status, respectively [19]. Although the association between BMI and the incidence of infection has been evaluated in several general population-based observational studies to date [19–28], the results were inconsistent and varied with the participants’ characteristics. Furthermore, no previous studies have focused on patients who are immunocompromised due to immunosuppressive treatment, including those with AAV. Therefore, the clinical impact of BMI on the incidence of infection in AAV patients receiving immunosuppressive therapy remained unknown.

In the present study, we hypothesized that older adults with AAV and low BMI would be at a higher risk of developing infection. To evaluate this hypothesis, we retrospectively investigated the association between underweight status at diagnosis of MPA and subsequent occurrence of severe infection in older adults with MPA in a single-center cohort in Japan.

## Methods

### Study population

**Fig. 1:**
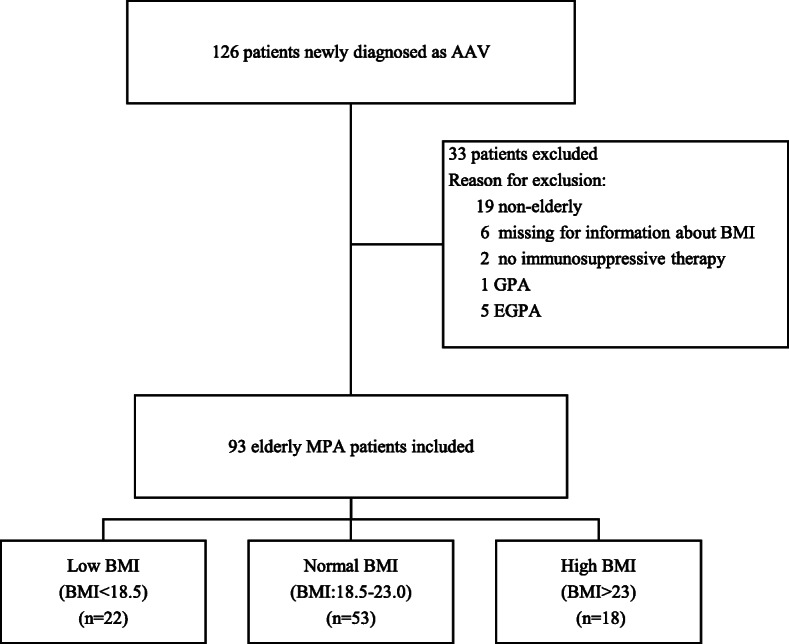
Flow diagram of the patient selection

The present study included 126 adult patients diagnosed with AAV, including GPA, MPA, and EGPA, at Nephrology and Rheumatology centers in the Aichi Medical University in Japan between 2004 and 2018. The AAV classification algorithm framed by the European Medicines Agency was applied in this study [29]. After excluding 33 (26.2%) aged < 65 years (*n* = 19), those who were not on immunosuppressive therapy (*n* = 2), those diagnosed with GPA (n = 1) or EGPA (*n* = 5), and those with missing BMI data (*n* = 6), 93 (73.8%) older adults with MPA who received immunosuppressive therapy were included in the present study (Fig. 1).

The study protocol was approved by the Ethics Committees of the Aichi Medical University (approval number 2018-H350, date November 3, 2019). The need to obtain patients’ informed consent was waived due to the retrospective nature of the study.

#### Data collection

Data collected retrospectively from patients’ medical records at the start of immunosuppressive therapy has been described in our previous study [30]. Briefly, the following baseline characteristics were collected: age, sex, BMI, body weight loss > 10% within 6-months before the diagnosis, lymphocyte count, serum creatinine level, serum albumin level, C-reactive protein level, serum IgG level, presence of diabetes mellitus, the Birmingham Vasculitis Activity Score (BVAS) 2003 [31], the 2009 Five-Factor Score (FFS) [32], organ involvement, and levels of anti-myeloperoxidase (MPO) and anti-proteinase 3 (PR3) antibodies. We also collated details of each patient’s immunosuppressive treatment including induction immunosuppressive therapy, methylprednisolone pulse therapy (0.5 or 1.0 g/day for 3 consecutive days), intravenous cyclophosphamide (CYC) or rituximab (RTX) use, and maintenance therapy with oral CYC, azathioprine (AZA), methotrexate, and/or RTX. Furthermore, we obtained details of the immunosuppressive treatment regimen such as the point dose of prednisolone (PSL) (mg/day), cumulative dose of PSL (mg/day), and use of any other immunosuppressants at 3, 6, 12, and 24 months from baseline.

We tested serum samples for MPO and PR3 ANCA by direct antigen-specific enzyme-linked immunosorbent assays, using serial serum dilutions, as described in an earlier study [29]. The samples were diluted 1:500 (Nipro Medical Corporation, Osaka, Japan) or 1:101 (Medical and Biological Laboratories Co., Ltd., Nagoya, Japan) times, as applicable.

### Outcomes

The primary parameter of interest was the BMI at the diagnosis of AAV. BMI classification was performed according to the World Health Organization Asian Standard [28], with underweight, normal weight, overweight, obesity I (moderate obesity), and obesity II (severe obesity) categories including patients with BMI values of < 18.5, 18.5–22.9, 23.0–24.9, 25.0–29.9, and ≥ 30 kg/m^2^, respectively. Considering the small number of patients categorized into overweight, obesity I, and II classes, we combined them into a single “high BMI” group. Thus, we finally stratified all patients into three categories of low (BMI < 18.5 kg/m^2^), normal (BMI, 18.5–23 kg/m^2^), and high (BMI >  23 kg/m^2^) BMI.

The main outcome of interest was development of severe infection, which was defined in our previous study [30] as requiring hospitalization for any cause. Similarly, remission was defined as the absence of clinical signs and symptoms of active vasculitis (BVAS = 0) for > 2-months, and relapse was defined as presence of clinical signs of vasculitic activity in any organ system after a period of remission, followed by an increase in requirement of corticosteroid dose and/or add-on use of immunosuppressive agents, as previously described [30, 33]. Data of other outcomes, including information about end-stage renal disease requiring dialysis, death, and hospitalization due to causes other than infection, were also collected. Patients were followed up until December 2019, and data were censored at death or on the last day of follow-up examination in our hospital during this period, as applicable.

### Statistical analyses

Baseline patient characteristics were summarized according to the three pre-defined BMI categories and were presented as percentages for categorical variables and as medians (interquartile ranges) for continuous variables with both normal and skewed distributions.

The associations of BMI with outcomes were assessed using univariate and multivariate Cox proportional hazard (CPH) models. The multivariate models were adjusted for potential confounding factors including age, sex, lung involvement, concurrent diabetes mellitus, serum creatinine level, use of methylprednisolone therapy, and BMI (low, normal, and high BMI), based on clinical experience and theoretical considerations. Furthermore, we assessed the above relationship using a Fine–Gray proportional subdistribution hazard model [34]. The proportional hazard assumption for covariates was tested using scaled Schoenfeld residuals. The Kruskal-Wallis test was used to evaluate the significance of intergroup differences for continuous variables. Categorical variables are expressed as percentages and were compared using the Pearson’s chi-square test. The cumulative probability of the development of the first severe infection was calculated using the Kaplan–Meier method and the log-rank test.

The level of statistical significance was set at *P* <  0.05. All statistical analyses were performed using JMP version 14.0.0 (SAS Institute, Cary, NC, USA), SAS statistical software version 9.4 (SAS Institute, Inc., Cary, NC), and STATA version 13.0 (StataCorp LP, College Station, TX, USA) software.

## Results

### Clinical characteristics

Baseline characteristics stratified as per the three BMI categories are shown in Table 1. The present cohort included 22 (23.7%), 53 (57.0%), and 18 (19.4%) patients with BMI < 18.5, 18.5–23.0, and >  23.0 kg/m^2^, respectively. On comparing baseline characteristics among these three groups, the proportion of patients with > 10% unintentional body weight loss within 6-months before the diagnosis of AAV, was significantly higher in the low BMI group than in the high BMI group (*P* = 0.016). No significant difference was observed among the three groups in other baseline characteristics.

**Table 1:** Clinical characteristics of 93 patients with MPA

	Low BMI (< 18.5) (*n* = 22)	Normal BMI (18.5–23.0) (*n* = 53)	High BMI (> 23) (*n* = 18)	*P* value
**Baseline characteristics**				
Age (years)	78 (70–81)	73 (69–78)	75 (69–78)	0.455
Male sex	15 (68.2)	27 (50.9)	8 (44.4)	0.267
BMI (kg/m^2^)	17.9 (17.0–18.3)	21.0 (20.0–22.1)	24.5 (23.7–26.4)	< 0.001
Body weight loss > 10% within 6 months before diagnosis	12 (54.5)	24 (45.3)	4 (22.2)	0.016
lymphocyte count (/μL)	964 (792–1101)	1008 (779–1336)	1120 (935–1367)	0.221
Serum creatinine level (mg/dL)	1.6 (1.0–6.5)	1.8 (0.9–3.8)	1.3 (0.6–2.3)	0.212
Serum albumin level (mg/dL)	3.0 (2.6–3.2)	2.8 (2.4–3.3)	3.0 (2.4–3.6)	0.739
Serum IgG level (mg/dL)	1826 (1590–1967)	1818 (1602–2019)	1760 (1346–2023)	0.583
CRP level (mg/dL)	6.5 (2.3–11.1)	3.6 (1.2–9.2)	5.8 (0.3–13.2)	0.379
Diabetes mellitus	7 (31.8)	9 (17.0)	4 (22.2)	0.362
Antibody				0.122
MPO-ANCA	22 (100)	53 (100)	17 (94.4)	
PR3-ANCA	0 (0)	0 (0)	1 (5.6)	
BVAS	15 (12–17)	14 (11–16)	14 (12–14)	0.591
FFS	2 (2–3)	2 (2–3)	2 (2–3)	0.618
Organ involvement				
General	22 (100)	53 (100)	17 (94.4)	0.122
Cutaneous	3 (13.0)	1 (1.8)	2 (10.5)	0.111
Ear nose and throat	4 (18.2)	14 (26.4)	5 (27.8)	0.713
Chest	6 (27.3)	20 (37.7)	5 (27.8)	0.584
Nodules or cavities	0 (0)	0 (0)	0 (0)	
Pleural effusion / pleurisy	0 (0)	2 (3.8)	0 (0)	
Endobronchial involvement	0 (0)	0 (0)	0 (0)	
Infiltrate	6 (100)	15 (75.0)	4 (80.0)	
Alveolar hemorrhage	0 (0)	3 (5.7)	1 (5.6)	
Cardiovascular	0 (0)	0 (0)	0 (0)	0.000
Abdominal	1 (4.6)	1 (1.9)	0 (0)	0.603
Renal	19 (86.4)	42 (79.3)	14 (77.8)	0.733
HD requirement at MPA diagnosis	5 (22.7)	1 (1.9)	2 (11.1)	0.013
Nervous system	4 (18.2)	8 (15.1)	4 (22.2)	0.779
Induction immunosuppressive therapy				
mPSL pulse therapy	11 (50.0)	25 (47.2)	8 (44.4)	0.940
Intravenous cyclophosphamide	3 (13.6)	4 (7.6)	1 (5.6)	0.608
Rituximab	0 (0)	3 (5.7)	2 (11.1)	0.298
Maintenance immunosuppressive therapy				0.381
Glucocorticoidmonotherapy	16 (72.7)	38 (71.7)	13 (72.2)	
Oralcyclophosphamide	0 (0)	1 (1.9)	1 (5.6)	
zathioprine	5 (22.7)	12 (22.6)	2 (11.1)	
Methotrexate	0 (0)	0 (0)	0 (0)	
Mizoribine	1 (4.6)	0 (0)	0 (0)	
Rituximab	0 (0)	2 (3.8)	2 (11.1)	
Outcomes				
Remission	18 (81.8)	48 (90.6)	16 (88.9)	0.562
Relapse	7 (38.9)	17 (35.4)	6 (37.5)	0.963
Severe infection	14 (63.6)	13 (24.5)	2 (11.1)	< 0.001
HD	8 (36.4)	11 (20.8)	4 (22.2)	0.348
Death	7 (31.8)	13 (24.5)	2 (11.1)	0.030
Infection	7 (100)	6 (46.2)	1 (50.0)	
Vasculitis	0 (0)	3 (23.1)	0 (0)	
Malignancy	0 (0)	2 (15.4)	0 (0)	
Cardiovascular	0 (0)	0 (0)	1 (50.0)	
Unknown	0 (0)	2 (15.4)	0 (0)	
Observation period (months)	18 (4–76)	17 (6–47)	32 (13–53)	0.828

Continuous data are presented as medians (interquartile ranges), and categorical data are expressed as numbers (proportions)

*Abbreviations*: *BMI* body mass index, *MPO* myeloperoxidase, *PR3* proteinase-3 ANCA, *ANCA* anti-neutrophil cytoplasmic antibody, *BVAS* Birmingham Vasculitis Activity Score, *FFS* Five-Factor Score, *MPA* microscopic polyangiitis, *mPSL* methylprednisolone, *HD* hemodialysis

### BMI and severe infection

**Fig. 2:**
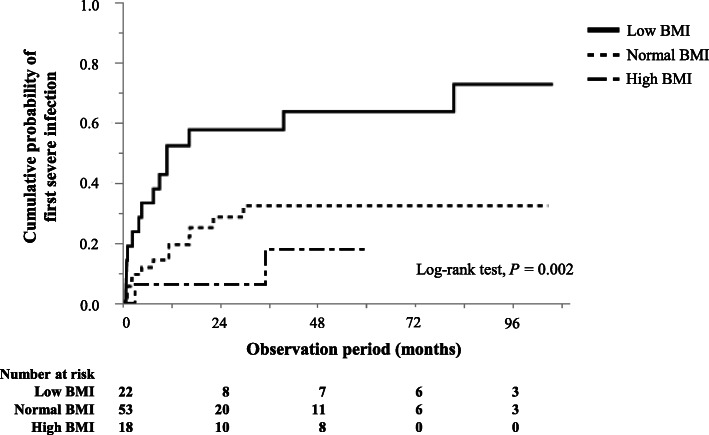
Cumulative probability of the first severe infection

During the study period, 34 severe infections occurred in 29 (31.2%) patients. Overall, 5 patients developed severe infections twice each, during the study period. The low BMI category was significantly associated with development of severe infection (*P* < 0.001). The cumulative probabilities of severe infection within 1, 2, and 5 years of diagnosis were, 0.52, 0.58, and 0.64 for those with low BMI; 0.20, 0.29, and 0.32 for those with normal BMI; and 0.06, 0.06, and 0.18 for those with high BMI, respectively. Occurrence of severe infection differed significantly according to the BMI category (log-rank, *P* = 0.002; Fig. 2). Causes of infection were miliary tuberculosis (*n* = 1), cytomegalovirus pneumonia (n = 1), influenza pneumonia (n = 1), bacterial pneumonia (*n* = 12), fungal pneumonia (*n* = 4), *Pneumocystis jiroveci* pneumonia (*n* = 2), acute colitis (n = 1), soft tissue infection (n = 1), infectious endocarditis (n = 1), methicillin-resistant *Staphylococcus aureus* bacteremia (n = 1), and acute pyelonephritis (n = 4).

A total of 90 (95.7%) patients had received prophylactic therapy with trimethoprim-sulfamethoxazole. The remaining 3 (3.2%) patients had to stop the drug due to allergic reactions. Of these, 2 patients eventually developed *Pneumocystis jiroveci* pneumonia.

### Predictors of severe infections

Predictors of severe infection were evaluated using age, sex, BMI, serum creatinine level, and use of methylprednisolone pulse therapy, as reported previously [15–18]. After univariate CPH analyses, older age, use of methylprednisolone pulse therapy, and low BMI were found to be statistically significant predictors of increased infection (Table 2). Multivariate analyses identified older age (adjusted hazard ratio [HR] = 2.02, 95% confidence interval [CI]: 1.14–3.52, per 10 years; *P* =  0.016), low BMI (< 18.5 kg/m^2^ compared with normal BMI [18.5–23.0 kg/m^2^], adjusted HR =  2.63, 95% CI: 1.11–6.19; *P* =  0.027), and use of methylprednisolone pulse therapy (adjusted HR = 2.48, 95% CI: 1.07–5.76; *P* =  0.034) as significant predictors of severe infection (Table 2).

**Table 2:** Predictors of the first severe infection in MPA

	Univariate model		Multivariate model	
	HR (95% CI)	*P*-value	HR (95% CI)	*P*-value
Age (per 10 years)	2.08 (1.16–3.67)	0.014	2.02 (1.14–3.52)	0.016
Male (vs. female)	1.11 (0.53–2.31)	0.782	1.22 (0.55–2.74)	0.624
Lung involvement	0.83 (0.38–1.84)	0.652	0.99 (0.39–5.76)	0.977
Serum creatinine (per 1.0 mg/dL)	1.10 (0.97–1.22)	0.124	1.05 (0.91–1.20)	0.504
Diabetes mellitus	1.17 (0.52–2.67)	0.703	0.77 (0.31–1.94)	0.582
mPSL pulse therapy	2.38 (1.10–5.12)	0.027	2.48 (1.07–5.76)	0.034
BMI groups				
Low BMI (< 18.5 kg/m^2^)	2.88 (1.35–6.16)	0.006	2.63 (1.11–6.19)	0.027
Normal BMI (18.5–23.0 kg/m^2^)	Reference		Reference	
High BMI (> 23.0 kg/m^2^)	0.43 (0.10–1.90)	0.265	0.42 (0.09–1.93)	0.266

Data are the HR, 95% CI, and *P* value from Cox proportional hazard regression analyses

The multivariate model was adjusted for baseline characteristics, including age, sex, lung involvement, serum creatinine level, diabetes mellitus, use of mPSL pulse therapy, and BMI groups (low, normal, and high BMI). “Normal BMI” was used as the reference category

*Abbreviations*: *BMI* body mass index, *mPSL* methylprednisolone, *MPA* microscopic polyangiitis, *HR* hazard ratio, *CI* confidence interval

Furthermore, these results did not differ with the application of Fine–Gray proportional subdistribution hazard models, (Supplemental Table 1), supporting a robust relationship between low BMI and severe infection.

PSL dose administration at baseline was the only variable found to be significantly higher in the high BMI group than that in the low and normal BMI groups (*P* < 0.001) (Table 3), indicating that differences in the intensity of immunosuppressive therapy between the three BMI groups might not influence the development of severe infection.

**Table 3:** Immunosuppressive treatment during the observation period

	Low BMI (< 18.5)	Normal BMI (18.5–23.0)	High BMI (> 23)	*P* value
Baseline	(*n* = 22)	(*n* = 53)	(*n* = 18)	
Prednisolone (mg/day)	30 (30–30)	30 (30–40)	34 (40–50)	< 0.001
3rd month	(*n* = 17)	(*n* = 46)	(*n* = 17)	
Prednisolone (mg/day)	13 (10–15)	13 (10–15)	13 (10–15)	0.808
Cumulative dose of prednisolone (g)	1.5 (1.5–1.6)	1.6 (1.4–1.8)	1.6 (1.4–1.9)	0.384
Use of immunosuppressants [n (%)]	1 (5.9)	6 (13.0)	2 (12.5)	0.309
6th month	(*n* = 15)	(*n* = 41)	(*n* = 15)	
Prednisolone (mg/day)	10 (8–10)	9 (8–10)	10 (7–10)	0.959
Cumulative dose of prednisolone (g)	2.6 (2.5–3.1)	2.5 (2.1–3.0)	2.4 (2.0–3.2)	0.360
Use of immunosuppressants [n (%)]	1 (6.7)	8 (19.5)	1 (6.7)	0.307
1st year	(*n* = 13)	(*n* = 36)	(*n* = 13)	
Prednisolone (mg/day)	6 (5–8)	7 (5–8)	8 (5–8)	0.892
Cumulative dose of prednisolone (g)	3.4 (3.1–4.1)	3.1 (2.7–43.9)	3.1 (2.7–3.8)	0.532
Use of immunosuppressants [n (%)]	0 (0)	8 (22.2)	1 (7.7)	0.110
2nd year	(*n* = 11)	(*n* = 26)	(*n* = 8)	
Prednisolone (mg/day)	5 (5–5)	5 (5–8)	5 (1–5)	0.267
Cumulative dose of prednisolone (g)	5.1 (4.5–5.7)	5.1 (4.3–6.0)	5.0 (3.0–6.4)	0.906
Use of immunosuppressants [n (%)]	1 (9.1)	11 (42.3)	1 (14.3)	0.081

Median (interquartile range), categorical values are expressed as numbers (proportions)

*Abbreviations*: *BMI* body mass index

Furthermore, we examined clinical characteristics of patients in the low BMI group, between those with and those without severe infections (Supplemental Table 2). Although indices for proportion of body weight loss (> 10% over prior 6-months), use of mPSL pulse therapy, and death were significantly higher in those who experienced severe infection than in those who did not, no other characteristics differed significantly between the two groups.

### Other outcomes

During the observation period, 18 (81.8), 48 (90.6), and 16 (88.9) patients in the low, normal, and high BMI group, respectively, achieved remission (*P* = 0.562). After achieving remission, 7 (38.9), 17 (35.4), and 6 (37.5) patients in the low, normal, and high BMI group, respectively, developed relapse (*P* = 0.963). During the observation period, 8 (36.4), 11 (20.8), and 4 (22.2) patients in the low, normal, and high BMI group, respectively, required permanent dialysis therapy (*P* = 0.348). A total of 22 patients died, including 7 (31.8), 13 (24.5), and 2 (11.1) patients in the low, normal, and high BMI group, respectively (*P* = 0.030). Overall, 14 (63.6%) patients died due to infection, including 7 (100), 6 (46.2), and 1 (50.0) from the low, normal, high BMI group, respectively.

## Discussion

The present retrospective and single-center study revealed that low BMI, at diagnosis of MPA, is significantly associated with subsequent development of severe infection in older adults in Japan. Our results suggested that malnutrition at diagnosis might increase vulnerability to infection during immunosuppressive treatment, especially in older adults. No previous study has focused on the clinical impact of underweight status on infection risk in older adults with MPA. Our results may provide a basis for identifying such patients who require more careful management to reduce risk of severe infection.

While the relationship between BMI and the risk of infection has been evaluated in several general population-based cohort studies, the findings were inconsistent and varied according to participants’ characteristics [20]. First, children and adolescents being underweight is considered a significant risk factor for infection in developing countries, probably reflecting the association with malnutrition and poor hygiene standards [21]. In contrast, data from industrialized countries suggest that the infection rate is increased in children and adolescents with obesity [22]. Several studies of adults from industrialized countries have suggested a U-shaped increase in the infection rate in both underweight and obese participants [23–25].

Thomas et al. studied 619 geriatric inpatients (≥75 years) and showed that both underweight (BMI < 20 kg/m^2^) and overweight (BMI > 28 kg/m^2^) status increased the risk of overall infection, including pneumonia, urinary tract infection (UTI), diarrhea, and others (incidence risk ratios: 1.84 [95% CI 1.40–2.42] for BMI < 20 kg/m^2^, 1.54 [95% CI 1.07–2.22] for BMI > 28 kg/m^2^) [26]. However, their study showed that while men experienced UTIs more frequently than women, older women are usually more susceptible to UTIs than their male counterparts [27]. Although the study ascribed this to the fact that more men than women were supplied with urinary catheters, their study participants might not reflect the general population, and the results should be interpreted with caution.

Additionally, a meta-analysis that included a large number of cohort studies, including 19,538 nursing home residents (median age, 84.3 years), revealed a higher risk of infection-related mortality (HR = 1.47 [95% CI 1.12–1.92]) in underweight individuals (BMI < 18.5 kg/m^2^), and a lower risk in overweight (BMI 25–29.9 kg/m^2^; HR = 0.70 [95% CI = 0.58–0.84]) and obese (BMI ≥ 30 kg/m^2^; HR = 0.63 [95% CI = 0.45–0.88]) participants than that in those having normal weight (BMI 18.5–25 kg/m^2^) [19].

A retrospective analysis of 66,820 clients (aged > 65 years) from Elderly Health Centres in Hong Kong revealed a U-shaped relationship between BMI and influenza-associated mortality in individuals stratified according to BMI, with HR of 1.081 (95% CI 1.013–1.154), 1.047 (1.012–1.084), 0.981 (0.936–1.028), 1.018 (0.980–1.058), and 1.062 (0.972–1.162) associated with underweight (BMI <  18.5 kg/m^2^), normal weight (BMI 18.5–22.9 kg/m^2^), overweight (BMI 23.0–24.9 kg/m^2^), obese I (BMI 25.0–29.9 kg/m^2^), and obese II (BMI ≥ 30 kg/m^2^) groups, respectively [28].

However, no previous studies had focused on the association between BMI and incidence of infection in immunocompromised patients who are on immunosuppressive treatment. In the present study, we focused on older adults with AAV who are at high risk for infection due to immunosuppressive therapy and found that underweight status at diagnosis was a significant predictor of risk of severe infection.

Furthermore, previous studies [19, 26, 27] did not assess change in body weight as an important factor for diagnosing malnutrition [35]. We found that the low BMI group frequently showed body weight loss preceding diagnosis of subsequent severe infections, suggesting that patients who were severely malnourished due to unintentional weight loss might be more vulnerable to immunosuppressive treatment, and may consequently be at an increased risk for developing severe infection.

Furthermore, in the present study, most infection-related events developed during the first year after AAV diagnosis in the low BMI group, a finding compatible with that of a previous study [8]. This result suggests that physicians should pay attention to development of infection, especially during the first year of treatment after diagnosis of AAV.

Although the precise mechanism of the influence of BMI on the immune system is unclear, undernourished subjects are found to show depleted leucocyte, lymphocyte, and T-cell counts, increased CD4/CD8 ratios, and decreased CD2/CD19 ratios [36, 37]. In addition, several factors with immunomodulatory effects, including physical activity [38], nutritional aspects (dietary composition or supplements) [39], and well-being [40] might modulate the risk of infection.

The present study has several limitations. First, due to the retrospective, observational nature of the present study, evaluation of clinical consequences of an altered immune response could not be fully adjusted for confounding factors, such as the underlying immune condition or comorbidities of each patient. In addition, various immunomodulatory factors as mentioned earlier, could not be assessed in the present study. Further studies should investigate the impact of these factors. Second, our study patients were older adults with MPA from Japan, and therefore the results may not be generalizable to young or middle-aged GPA patients from other geographic areas. Third, glucocorticoid monotherapy was frequently used by patients in the present study, a finding that corroborated with that of previous Japanese studies [41, 42] on infection risk in older adults with MPA. However, this practice pattern differs from that followed worldwide, wherein RTX or CYC are commonly used for remission and/or maintenance therapy [43]. Therefore, our results should be interpreted cautiously. Fourth, we had no data about vaccination status and regarding chronic nasal carriage of *Staphylococcus aureus* that might influence infection development. Further studies including these parameters should be undertaken. Fifth, BMI has several limitations including the inability to account for cachexic alterations such as loss or gain of fat-free mass. Our study could not evaluate the nutrition status more accurately using methods as such as bioelectrical impedance vector analysis, to identify cachectic and non-cachectic patients. Finally, we were unable to assess the effect of the overall BMI status throughout a patient’s lifetime, on the outcomes observed during the entire follow-up period. Importantly, the impact of the change in BMI after starting immunosuppressive treatment could not be assessed. We believe that potential preventive measures that focus on improving nutritional intake in these high-risk patients should be evaluated in further studies.

## Conclusions

The present study identified a lower BMI as a significant predictor of the risk of severe infection in older adults with MPA. This suggests that physicians should closely follow-up older adults with MPA and low BMI to monitor them for the development of infections.

## Supplementary Information

**Additional file 1: Supplemental Table 1**. Predictors of first severe infection in MPA.

**Additional file 2: Supplementary Table 2**. Comparison between MPA patients with low BMI with and without severe infection.

**Additional file 3.**

## Data Availability

All data generated or analyzed during this study are included in this published article and its supplementary information files. All data were fully anonymized (Supplemental Table 3).

## Abbreviations

AAV: Antineutrophil cytoplasmic antibody-associated vasculitis
ANCA: Antineutrophil cytoplasmic antibody
AZA: Azathioprine
BMI: Body mass index
BVAS: Birmingham Vasculitis Activity Score
CI: Confidence interval
CYC: Cyclophosphamide
EGPA: Eosinophilic granulomatosis with polyangiitis
GPA: Granulomatosis with polyangiitis
HD: Hemodialysis
HR: Hazard ratio
MPA: Microscopic polyangiitis
MPO: Myeloperoxidase
mPSL: methylprednisolone
PR3: Proteinase 3
RTX: Rituximab
