# Supplementary material for: Association between body mass index and severe infection in older adults with microscopic polyangiitis: a retrospective cohort in Japan
Source: BMC Geriatr. 2021 Mar 9;21:171. doi: 10.1186/s12877-021-02123-y (PMC7942001; doi:10.1186/s12877-021-02123-y)
Supplement: Supplementary file 1 — Additional file 1: Supplemental Table 1. Predictors of first severe infection in MPA. [file 12877_2021_2123_MOESM1_ESM.docx]

**Supplemental Table 1**. Predictors of first severe infection in MPA

|  | Univariate model | |  | Multivariate model | |
| --- | --- | --- | --- | --- | --- |
|  | HR (95% CI) | *P*-value |  | HR (95% CI) | *P*-value |
| Age (per 10 years) | 2.08  (1.17–3.69) | 0.012 |  | 2.02  (1.16–3.54) | 0.014 |
| Male (vs. female) | 1.11  (0.53–2.33) | 0.782 |  | 1.22  (0.55–2.70) | 0.624 |
| Lung involvement | 0.83  (0.38–1.84) | 0.652 |  | 0.99  (0.39–2.48) | 0.977 |
| Serum creatinine (per 1.0 mg/dL) | 1.10  (0.98–1.24) | 0.105 |  | 1.05  (0.91–1.20) | 0.504 |
| Diabetes mellitus | 1.17  (0.52–2.66) | 0.703 |  | 0.77  (0.31–1.94) | 0.582 |
| mPSL pulse therapy | 2.38  (1.10–5.12) | 0.027 |  | 2.48  (1.07–5.76) | 0.034 |
| BMI groups |  |  |  |  |  |
| Low BMI (< 18.5 kg/m^2^) | 2.88  (1.35–6.16) | 0.006 |  | 2.63  (1.11–6.19) | 0.027 |
| Normal BMI (18.5‒23.0 kg/m^2^) | Reference |  |  | Reference |  |
| High BMI (> 23.0 kg/m^2^) | 0.43  (0.10–1.90) | 0.265 |  | 0.42  (0.09–1.93) | 0.266 |

Data are the HR, 95% CI, and *P* value from Fine–Gray proportional subdistribution hazard models.

The multivariate model was adjusted for baseline characteristics, including age, sex, lung involvement, serum creatinine level, diabetes mellitus, use of mPSL pulse therapy, and BMI groups (low, normal, and high BMI). “Normal BMI” was used as the reference category.

Abbreviations: BMI, body mass index; mPSL, methylprednisolone, MPA, microscopic polyangiitis; HR, hazard ratio; CI, confidence interval
