# Supplementary material for: Association between body mass index and severe infection in older adults with microscopic polyangiitis: a retrospective cohort in Japan
Source: BMC Geriatr. 2021 Mar 9;21:171. doi: 10.1186/s12877-021-02123-y (PMC7942001; doi:10.1186/s12877-021-02123-y)
Supplement: Supplementary file 2 — Additional file 2: Supplementary Table 2. Comparison between MPA patients with low BMI with and without severe infection. [file 12877_2021_2123_MOESM2_ESM.docx]

**Supplementary Table 2**. Comparison between MPA patients with low BMI with and without severe infection.

|  | Severe infection  (n = 14) | No severe infection  (n = 8) | *P* value |
| --- | --- | --- | --- |
| **Baseline characteristics** |  |  |  |
| Age (years) | 78 (73‒81) | 72 (66‒81) | 0.170 |
| Male sex | 9 (64.3) | 6 (75.0) | 0.604 |
| BMI (kg/m^2^) | 17.4 (16.3‒18.4) | 18.1 (17.8‒18.3) | 0.219 |
| Body weight loss > 10% within 6 months before diagnosis | 11 (78.6) | 1 (12.5) | 0.009 |
| lymphocyte count (/μL) | 1060 (746-1157) | 860 (807-995) | 0.357 |
| Serum creatinine level (mg/dL) | 1.6 (1.0‒6.4) | 2.6 (1.1‒8.2) | 0.682 |
| Serum albumin level (mg/dL) | 3.0 (2.5‒3.1) | 3.2 (2.7‒3.3) | 0.273 |
| Serum IgG level (mg/dL) | 1901 (1509‒2005) | 1796 (1639‒1878) | 0.339 |
| CRP level (mg/dL) | 6.5 (3.7‒13.5) | 6.3 (1.7‒10.7) | 0.488 |
| Diabetes mellitus | 4 (28.6) | 3 (37.5) | 0.665 |
| Antibody |  |  | 0.000 |
| MPO-ANCA | 14 (100) | 8 (100) |  |
| PR3-ANCA | 0 (0) | 0 (0) |  |
| BVAS | 15 (13‒17) | 13 (11‒22) | 0.864 |
| Organ involvement |  |  |  |
| General | 14 (100) | 8 (100) | 0.000 |
| Cutaneous | 1 (7.1) | 1 (12.5) | 0.674 |
| Ear nose and throat | 3 (21.4) | 3 (37.5) | 0.416 |
| Chest | 3 (21.4) | 3 (37.5) | 0.416 |
| Nodules or cavities | 0 (0) | 0 (0) |  |
| Pleural effusion / pleurisy | 0 (0) | 0 (0) |  |
| Endobronchial involvement | 0 (0) | 0 (0) |  |
| Infiltrate | 4 (28.6) | 3 (37.5) |  |
| Alveolar hemorrhage | 3 (5.7) | 1 (5.6) |  |
| Cardiovascular | 0 (0) | 0 (0) | 0.000 |
| Abdominal | 0 (0) | 1 (12.5) | 0.176 |
| Renal | 13 (92.9) | 6 (75.0) | 0.240 |
| HD requirement at MPA diagnosis | 3 (21.4) | 2 (25.0) | 0.848 |
| Nervous system | 3 (21.4) | 1 (12.5) | 0.601 |
| Induction immunosuppressive therapy |  |  |  |
| mPSL pulse therapy | 10 (71.4) | 1 (12.5) | 0.008 |
| Intravenous cyclophosphamide | 3 (21.4) | 0 (0) | 0.159 |
| Rituximab | 0 (0) | 0 (0) | 0.000 |
| Maintenance immunosuppressive therapy |  |  | 0.466 |
| Glucocorticoid monotherapy | 9 (64.3) | 7 (87.5) |  |
| Oral cyclophosphamide | 0 (0) | 0 (0) |  |
| Azathioprine | 4 (28.6) | 1 (12.5) |  |
| Methotrexate | 0 (0) | 0 (0) |  |
| Mizoribine | 1 (7.1) | 0 (0) |  |
| Rituximab | 0 (0) | 0 (0) |  |
| Outcomes |  |  |  |
| Remission | 11 (78.6) | 7 (87.5) | 0.601 |
| Relapse | 5 (45.5) | 2 (28.6) | 0.474 |
| HD | 4 (28.6) | 4 (50.0) | 0.315 |
| Death | 7 (50.0) | 0 (0) | 0.015 |
| Infection | 7 (100) | 0 (0) |  |
| Observation period (months) | 10 (3‒54) | 64 (14‒100) | 0.088 |

Continuous data are presented as a median (interquartile range), and categorical data are expressed as a number (proportion).

Abbreviations: BMI, body mass index; MPO, myeloperoxidase; PR3, proteinase-3 ANCA; ANCA, anti-neutrophil cytoplasmic antibody; AAV, anti-neutrophil cytoplasmic antibody-associated vasculitis; MPA, microscopic polyangiitis; mPSL, methylprednisolone; HD, hemodialysis;
